# Evaluation Study of Intraoperative Cytology Smear and Frozen Section of Glioma

**DOI:** 10.31557/APJCP.2020.21.10.3085

**Published:** 2020-10

**Authors:** Sarah Zulkarnain, Norhayati Yunus, Regunath Kandasamy, Ahmad Badruridzwanullah Zun, Anani Aila Mat Zin

**Affiliations:** 1 *Department of Pathology, School of Medical Sciences, Universiti Sains Malaysia, Health Campus, 16150 Kota Bharu, Kelantan, Malaysia. *; 2 *Department of Pathology, Hospital Raja Perempuan Zainab II, 15586 Kota Bharu, Kelantan, Malaysia.*; 3 *Department of Neuroscience, School of Medical Sciences, Universiti Sains Malaysia, Health Campus, 16150 Kota Bharu, Kelantan, Malaysia.*; 4 *Department of Community Medicine, School of Medical Sciences, Universiti Sains Malaysia, Health Campus, 16150 Kota Bharu, Kelantan, Malaysia.*

**Keywords:** Diagnostic accuracy, cytology smear, frozen section, glioma

## Abstract

**Objective:**

Glioma is the commonest primary malignant brain tumour. Diagnosis is made based on cytology smear, frozen section and histopathological examination. Intraoperative pathological diagnosis using either cytology smear, frozen section or combination of both, plays a crucial role in patient’s future management and prognosis. This study aims to determine the accuracy of cytology smear and frozen section in glioma, and to compare the difference between both techniques.

**Methods:**

A cross-sectional study was conducted involving 22 cases of glioma diagnosed intraoperatively from January 2013 until August 2019 in Hospital Universiti Sains Malaysia. The selected tissues were processed for cytology smear and frozen section. The remaining tissues were proceeded for paraffin section. The diagnosis was categorized as either low-grade or high-grade glioma based on cellularity, nuclear pleomorphism, mitotic count, microvascular proliferation and necrosis. The sensitivity and specificity of frozen section and cytology smears were determined based on paraffin section being as the gold standard. The accuracy of both techniques was compared using statistical analysis.

**Results:**

The overall sensitivity and specificity of cytology smear were 100% and 76.9%, respectively. Meanwhile, the sensitivity and specificity of frozen section were 100% and 84.6%. There was no significant difference in diagnostic accuracy between cytology smear and frozen section in glioma (p>0.05).

**Conclusion:**

Cytology smears provides an alternative method for frozen section due to good cellularity and morphology on smear. Cytology smear is rapid, inexpensive, small amount of tissue requirement and less technical demand. This finding may benefit to the hospital or treatment centres where frozen section facility is unavailable.

## Introduction

Glioma is a primary central nervous system tumour arises in glial tissue and it can be further divided as astrocytoma, oligodendroglioma, oligoastrocytoma or ependymoma. Worldwide, glioma is the most common primary intracranial malignancy. Glioma accounts for 75% of malignant brain tumours, and more than half out of these are glioblastomas (Ostrom et al., 2017). Intraoperative histopathological diagnosis plays a crucial role in optimizing the surgical procedure and determining the treatment plan of the patient. In certain situation, it can detect an unexpected lesion that cannot be determined by clinical or radiological imaging. 

Frozen section has been an established intraoperative histopathological evaluation worldwide since it was first introduced in 1891. Subsequently, cytology smear was introduced later and there was an increased usage of this technique for supplementing or replacing the use of frozen section technique (Jaiswal et al., 2012).

Cytology smear techniques include squash smears and touch imprints are rapid, less expensive, technically much easier and requires only a small piece of tissue for each slide with high percentage of accuracy between 84.9%-95.36% (Rao et al., 2009; Mitra et al., 2010; Sharma et al., 2011; Nanarng et al., 2015; Chand et al., 2016; Patil et al., 2016; Sharifabadi et al., 2016). It also provides an alternative method when facilities for frozen section are limited (Ahmed et al., 2015). 

Meanwhile, frozen section technique is rapid and able to demonstrate the architectural characteristic however the major drawback of using this technique is freezing artefact. Besides that, this technique requires trained staff and an expensive equipment.

## Materials and Methods

*Case and sample selection*

This study was a cross-sectional study conducted at Hospital Universiti Sains Malaysia (USM) involving 22 cases of glioma diagnosed intraoperatively within period of 6 years and 7 months from January 2013 until August 2019. The retrospective samples were obtained from the archived Lab Information System (LIS). Frozen section, cytology smear and paraffin slides were retrieved from the Hospital USM lab. The demographic data and neuroimaging findings were collected through patient’s pathology reports. 

For prospective samples, the samples were obtained from the surgeon intraoperatively. The selected tissues were processed for cytology smear and frozen section. The remaining tissues were proceeded with paraffin section. 22 cases fulfilled the inclusion criteria. Patients diagnosed with brain tumour other than glioma or unavailability of cytology smear and/or frozen section and/or paraffin section were excluded.

The diagnosis was categorized as either low grade or high grade glioma according to St. Ann-Mayo grading system that include cellularity, nuclear pleomorphism, mitotic count, microvascular proliferation and necrosis. WHO grade I and II were grouped as low grade glioma while WHO grade III and IV were consider as high grade glioma. The sensitivity and specificity of the cytology smear and frozen section were determined on paraffin section being as the gold standard. The accuracy of both techniques was compared using statistical analysis.

 The sample size was calculated using the estimation of sensitivity and specificity sample size calculator by Dr Lin Naing@Mohd Ayub Sadiq from School of Dental Sciences, USM. The estimated sample size to answer the objectives was 48 calculated based on the expected sensitivity and specificity were 100% and 86.21% respectively (Ahmed et al., 2015). This study was approved by the Human Research Ethics Committee, USM with the reference USM/JEPeM/18010083.

*Cytology smears*

The cytology smear’s methods applied were touch imprint and crush smear. In touch imprint, the fresh unfixed specimen was gently touched on a labelled glass slide using a forceps. Fresh unfixed specimen was gently crushed on a labelled glass slide by a second slide held at right angle during preparation of crush smear. Both air dried smears and 95% ethyl alcohol fixed smears were stained using hematoxylin and eosin (H & E) stains. 

*Frozen section*

The specimens were processed in cryostat machine at -22^o^ celcius to -26^o^ celcius. Initially, the specimens were described and measured. Then, they were placed on the chuck which has spread with cryo compound. The chuck was inserted to the location hole and oriented accordingly. Subsequently, the specimens were trimmed in order to obtain a plane parallel surface. Sectioning started at approximately 5-8 µm in thickness, then the sectioning thickness will be decrease to the required value. Finally, sectioned slides were stained using rapid H & E stain and for slide mounting. The remaining tissues were fixed in formalin for paraffin section preparation and subsequently stained for H & E stain.

*Statistical analysis*

The clinicopathological information were analysed using the descriptive statistic. The sensitivity, specificity, positive predictive value and negative predictive value were calculated using specific formula for each value. Mc Nemar test was used to determine the significant difference in the proportion of accuracy between cytology smear and frozen section in glioma from the same specimens using Statistical Package for Social Sciences (SPSS) software version 20.

## Results

*Clinicopathological data*

The clinicopathological data for glioma is presented in Table 1. A total of 22 glioma cases were included in this study. The median age of glioma cases diagnosed in Hospital USM by cytology smear and frozen section was 31.7. The number of male and female patients diagnosed with glioma were almost equal, total of 12 female patients (54.5%) and 10 male patients (45.5%). All the patients were Malay. The commonest presenting symptoms were headache whereby 54.5% of the glioma patients presented with this complaint followed by hemiplegia, 45.5%; vomiting, 22.7%; seizure, 18.2% and 9.1% each for blurring of vision, speech disturbance, facial weakness and unsteady gait. The common site of glioma occurrence was frontal (40.9%) followed by temporal (9.1%), cerebellum (9.1%), basal ganglia (9.1%), thalamus (9.1%) with parietal, ventricular, pons, corpus callosum and pineal region that account for 4.5% each. 

**Figure 1:**
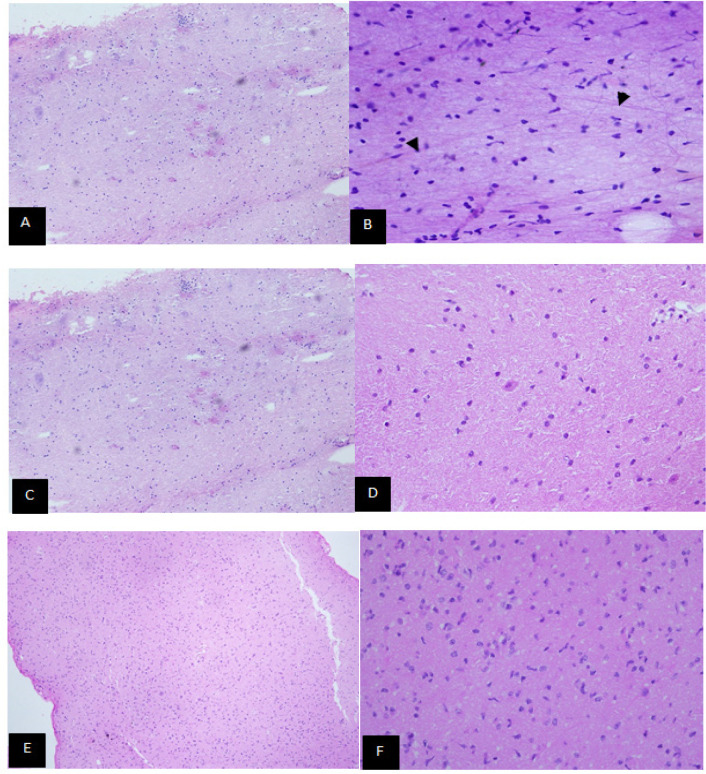
(A) Cytology smear for low grade glioma. At x200 magnification: Smears are of low cellularity. (B) At x400 magnification: Smears composed of uniform tumour cells. Fine fibrillary glial tissue is seen in the background (arrowhead). (C) Frozen section of low grade glioma at x100 magnification and x400 magnification in (D) which show low cellularity with absent of significant nuclear atypia. Paraffin section of low grade glioma at x100 magnification in (E) show tumour tissue with low cellularity and x400 magnification in (F), the tumour cells are generally uniform

A total of 13 low grade glioma and 9 high-grade glioma cases were diagnosed from paraffin section. Meanwhile, 11 cases each for low grade and high grade glioma were diagnosed based from frozen section. Cytology smears diagnosed 10 cases of low grade glioma with 12 cases of high grade glioma. The morphologies of the examined slides are shown in Figure 1 and 2.

The distribution of glioma is depicted in Table 2. Low and high grade glioma were frequently diagnosed after two decades of life with both grades having 8 out of 22 cases (36.4%). Low grade glioma was common among male (40.9%) in comparison with high grade glioma which was common in female (27.3%). Majority of the low grade and high grade glioma arise from the frontal region of the cerebral hemisphere (18.2 % in low grade and 22.7% in high grade glioma).

*Sensitivity and specificity of cytology smear and frozen section*

The overall sensitivity of cytology smears was 100% while only 10 out of 13 low- grade glioma cases were able to detect giving the overall specificity of 76.9%. The positive predictive value and negative predictive value for frozen section were 75% and 100%, respectively.

The overall sensitivity of frozen section was 100% whereby frozen section able to detect all 9 high grade glioma cases. Frozen section able to detect only 11 low grade glioma cases from total of 13 low grade glioma cases giving the overall specificity of 84.6%. The positive predictive value and negative predictive value for frozen section were 81.8% and 100%, respectively. 

*Difference in the proportion of accuracy between cytology smear and frozen section*

There are 19 glioma cases that were correctly graded by cytology smear and frozen section. On the other hand, grading was inaccurate in 2 of the cases by both methods. There is one glioma case that was correctly graded by frozen section in which cytology smear failed. Based on Mc Nemar test, there was no significant difference in the proportion of accuracy between cytology smear and frozen section, giving p value of >0.05 (Table 3). 

**Table 1:** Clinicopathological Distributions of the Glioma Cases (n=22)

Variables	Mean (SD)	N (%)
Age		
0 to 14 years old		6 (27.3)
15 to 19 years old	31.7 (19.1)	0 (0.0)
20 to 60 years old		16 (72.7)
Gender		
Male		12 (54.5)
Female		10 (45.5)
Race		
Malay		22 (100)
Others		0 (0.0)
Clinical Symptoms		
Headache		12 (54.5)
Hemiplagia		10 (45.5)
Vomiting		5 (22.7)
Seizure		4 (18.2)
Blurring of vision		2 (9.1)
Speech disturbance		2 (9.1)
Facial weakness		2 (9.1)
Unsteady gait		2 (9.1)
Tumor Location		
Frontal		9 (40.9)
Temporal		2 (9.1)
Cerebellum		2 (9.1)
Basal ganglia		2 (9.1)
Parietal		1 (4.5)
Ventricular		1 (4.5)
Thalamus		2 (9.1)
Pons		1 (4.5)
Corpus Callosum		1 (4.5)
Pineal region		1 (4.5)
Paraffin		
Low grade		13 (59.1)
High grade		9 (40.9)
Frozen		
Low grade		11 (50.0)
High grade		11 (50.0)
Cytology		
Low grade		10 (45.5)
High grade		12 (54.5)

**Table 2:** Distribution of Tumour Grade Based on Age, Gender and Tumour Location (n=22)

	Paraffin diagnosis, n (%)	
Variables	Low Grade	High grade
Age		
0 to 14 years old	5 (22.7)	1 (4.5)
15 to 19 years old	0 (0.0)	0 (0.0)
20 to 60 years old	8 (36.4)	8 (36.4)
Gender		
Male	9 (40.9)	3 (13.6)
Female	4 (18.2)	6 (27.3)
Tumor Location		
Frontal	4 (18.2)	5 (22.7)
Temporal	2 (9.1)	0 (0.0)
Cerebellum	2 (9.1)	0 (0.0)
Basal ganglia	1 (4.5)	1 (4.5)
Parietal	0 (0.0)	1 (4.5)
Ventricular	1 (4.5)	0 (0.0)
Thalamus	0 (0.0)	2 (9.1)
Pons	1 (4.5)	0 (0.0)
Corpus Callosum	1 (4.5)	0 (0.0)
Pineal region	1 (4.5)	0 (0.0)

**Table 3:** The Difference in Proportion of Accuracy between Cytology Smear and Frozen Section (n=22)

Variables	Cytology Smear, n (%)		
	Accurate	Not Accurate	P value*
Frozen Section			
Accurate	19 (86.4)	1 (4.5)	1
Not accurate	0 (0.0)	2 (9.1)	

*Mc Nemar Test, significant when p value <0.05

## Discussion

Glioma is the commonest brain tumour in United States and Malaysia (Ostrom et al., 2017; Azizah et al., 2016). Glioma also has been the predominant neoplastic lesion in most of the studies (Pawar et al., 2009; Rao et al., 2009; Mitra et al., 2010; Cheunsuchon et al., 2014; Shrestha et al., 2014; Chand et al., 2016; Amraei et al., 2017). This tumour possesses distinct clinicopathological characteristic with regards to the age, sex and tumour location. 

In our study, both genders were almost equally affected. In contrast to the latest Malaysian National Cancer of registry from 2007-2011, brain tumour was predominant in male. It was the 11th most frequent tumour in male (Azizah et al., 2016). There was a slight higher percentage of cases reported in female, 57.9% reported by The Central Brain Tumor Registry of the United States (CBTRUS) from 2010 to 2014. However, malignant brain tumour cases were common in male, 55.4% compared to females, 44.6%. Among these, glioma specifically glioblastoma was the commonest malignant brain tumour (Ostrom et al., 2017). According to Danish Neuro-Oncology Registry (DNOR) 2009 to 2014, there was slight male predominance, with overall male:female ratio of 3:2 (Rasmussen et al., 2017). 

Based on Malaysian National Cancer of Registry from 2007-2011, higher incidence rate of brain and nervous system cancer was observed in Chinese followed by Malay and Indian (Azizah et al., 2016). However, Malay population predominates in our study. The finding was representative of Kelantan population which composed of 99% of Malay population followed by Chinese, Indian and minority of Siamese population.

In general, glioma can affect wide range of age from paediatric to older age group. In our study, the mean age was 31.7. Both low grade and high grade glioma occurred after two decades of life. Glioma with older age group were observed in United States with median age of 59 years old (Ostrom et al., 2017). All of our paediatric cases were diagnosed as low grade glioma in contrast with high grade glioma which only occurred at older age group. These findings were agreed by other studies whereby Danish Neuro-oncology stated that the mean age increased with the tumour grading of glioma (Rasmussen et al., 2017). Pilocytic astrocytoma is common in younger age group with age adjusted incidence rate of 0.89 per 100 000 population and the incidence decreases as the age advanced (Ostrom et al., 2017). On the other hand, glioblastoma is common among older age group and the incidence increase as the age recline. 

The distribution of tumour site depends on the tumour grading. In general, glioma occur at frontal (23.7%), temporal (17.4%), parietal (10.5%), and occipital (2.7%) of the cerebral hemispheres (Ostrom et al., 2017). Similar locations were observed in our study where frontal and temporal being the commonest tumour location. Based on grading, high grade glioma is commonly occurred at the frontal lobe. Some studies reported that Glioblastoma (IDH-mutant), WHO grade IV; anaplastic astrocytoma, WHO grade III and anaplastic oligodendroglioma, WHO grade III predominantly located in frontal region (Lai et al., 2011; Louis et al., 2016).

Neurosurgical, neuroimaging and neuropathological experts play a crucial role in diagnosing and managing patient with glioma. The pathologist must be well-experienced and knowledgeable in handling glioma cases. Before treatment of patient is commenced, appropriate diagnosis must be made. Histological examination remained the gold standard to diagnosed glioma (Brat et al., 2008). Thus, the principle role of pathologist is to provide the accurate diagnosis from the brain tissue sent by the neurosurgeon in order to determine the best treatment for the patient.

Certain indications necessitate the need for intraoperative diagnosis especially in situation whereby neuroimaging unable to give conclusive diagnosis or disagreement between clinical presentation and neuroimaging (Roessler et al., 2002; Nanarng et al., 2015; Sharifabadi et al., 2016). Other reasons include guidance for the surgeon on the extent of surgery, to determine the adequacy of tissue sample especially in cases with extensive areas of necrosis, to differentiate between neoplastic and nonneoplastic lesion as well as tumour grading. 

The laboratory techniques used during intraoperative diagnosis are either cytology smears, frozen section or both depends on the availability of resources. Thus, determination of diagnostic accuracy of both techniques in glioma, understanding the strength and limitation in addition to clinicopathology characteristic are of major importance to ensure the best and optimum management of patient with glioma. Accuracy depends on complete clinical and radiological findings, good sampling, technical performance and competency of the pathologist (Khamechian et al., 2012; Kumar et al., 2013; Obeidat et al., 2016; Patil et al., 2016).

Frozen section has been the established and preferred modalities for intraoperative diagnosis of glioma. Histology architectural detail and cytomorphology are best visualized by frozen section. The major drawback of frozen section is freezing artefact. On the other hand, cytology smear is rapid, low-cost, effortless, does not required experienced staff and expensive equipment. This technique is very useful when limited tissue sample is provided by the neurosurgeon especially in the recent advancement of stereotactic biopsy. Thus, enough tissue is available for further paraffin section. 

It said that cytology smear and frozen section complement each other as each technique overcome the disadvantages of the other technique. Cytology smears are best in diagnosing glioma due to soft consistency and high water content of glioma tissue (Chand et al., 2016). High water content can cause marked freezing artefact in frozen section that may alter the architecture and cytomorphology of the tumour. Moreover, cytology smear can be smeared easily and displayed good cytoarchitectural detail. Cytology smear visualized better tumour matrix specifically glial fibrillary background in gliomas that will give cotton-like appearance. It can differentiate whether the matrix is reactive process or high grade glioma based on the quality of the fibrillary filament. Fine filament is present in reactive process while coarse filament as in high grade glioma (Joseph, 2007).

One of the features in pilocytic astrocytoma which is Rosenthal fibers are well appreciated in cytology smear (Louis et al., 2016). Other than that, the nuclear and cytoplasmic details are better observed by cytology smear (Kumar et al., 2013). Cytology smear maintained the nuclear and cytoplasmic characteristic of the tumour cells. Unlike in frozen section where freezing artefacts will cause crenated nuclei (Joseph, 2007). This will lead to difficulties in making final diagnosis as nuclear characteristic is hardly appreciated.

Cytology smear techniques include touch imprint and crush smear. From our study, touch imprint is very helpful in high cellularity tumour as crush smear introduced marked crush artefact which hinder the true cytoarchitectural details. The thickness of the smear produced was even and the architectural details such as rosettes formation, Rosenthal fibres and microcystic areas were maintained. These architectures may be demolished if high pressure is applied in crushed smear. These findings are supported by Sharma et al., (2011) and Sharifabadi et al., (2016). Touch imprint is useful in case of lymphoma as the tumour is easily detached and evenly spread (Nanarng et al., 2015). However, touch imprint is not recommended for low cellularity tumour as this will lead to low-cellular yield which cause limited availability of the sample for analysis. 

We graded the glioma based on St. Anne-Mayo, 4-tiered grading system that includes tumour cellularity, presence of nuclear atypia, endothelial proliferation, increase mitosis and necrosis. This grading system are widely used in previous studies (Brat et al., 2008; Louis and von Deimling, 2017). In low grade glioma, the cellularity is low and the cells display uniform nuclei. Minimal anisocytosis is acceptable which can be observed in some cases. In our study, angiogenesis may be visualized in low grade glioma but the vessels were in capillary-sized vessels. However, none of low grade glioma in our study exhibit vascular endothelial proliferation which is observed in high grade glioma case. Occasional mitosis (not more than 1) is allowed specifically in grade II (Brat et al., 2008). 

In contrast, high grade glioma required at least 2 or more features to diagnose according to the St. Anne-Mayo, 4-tiered grading system (Roessler et al., 2002). The cellularity is mostly high with significant nuclear atypia. The criteria of nuclear atypia include nuclear pleomorphism, enlarged hyperchromatic nuclei, coarse chromatin and prominent nucleoli in some of the tumour cells. Multiple large vessels proliferation with vascular endothelial proliferation, increase in mitosis and necrosis are visualized. In our study, all high grade glioma cases showed high cellularity and marked nuclear atypia. Majority of the cases contained multiple large vessels proliferation with endothelial proliferation. The density of vascular proliferation is more pronounced in comparison with low grade glioma. Three of the cases displayed prominent necrosis. Mitosis is hardly identified in which only one case noted to have mitosis. The mitosis is best identified by paraffin section. 

In our current study, the overall diagnostic accuracy of cytology smear was 86.3%. The result is comparable with other studies with the accuracy ranging from 84.9-91.25% (Mitra et al., 2010; Chand et al., 2016; Patil et al., 2016; Samal et al., 2017). Nineteen out of twenty-two cases were correctly graded. Three of the cases were over graded as high grade glioma as the paraffin section revealed low grade glioma. The contributing factors were marked crushed artefact and thickened smear. Crush artefact in crushed smear demonstrates anisonucleosis while thickened smear was misinterpreted as high cellularity. A study also experienced similar issues in a case of low grade oligoastrocytoma which was over diagnosed as high grade glioma (Nanarng et al., 2015). This highlighted the importance of good smearing technique to overcome the shortcomings of crushed smear. 

One of the cases diagnosed by cytology smear was over graded as high grade glioma due to misinterpretation of the density of vascular proliferation. This situation is significantly relevant in a case of oligodendroglioma where vascular proliferation is one of the characteristics (Chand et al., 2016). Vessels proliferation in the tumour is meant to provide adequate nutrition for the growth. Only vessels proliferation with endothelial proliferation is considered for diagnosis of high grade glioma. Majority of high grade glioma in our study exhibit large vessels proliferation. 

Pathologist encountered some difficulties in grading glioma due to its heterogeneity of the tumour (Pawar et al., 2009; Mitra et al., 2010; Chand et al., 2016). Different tumour site may reflect different characteristic. In areas, the tumour may reveal low grade area while high grade area is observed in other area. Cytology smear consumes small tissue sample that has raised the issue of representative sample and adequate tissue sampling by the neurosurgeon. Misdiagnosed occurred when wrong tissue site was sampled that do not reflect the true behaviour of the glioma. We encountered problem in diagnosing one of the tissue sample. The sample was non representative as the slide revealed only red blood cells. Good communication between the pathologist and neurosurgeon are mandatory to establish the accurate diagnosis. It is advisable for the pathologist to ask for further sample if the sample is not representative or inadequate. The sample must be adequate and representative of the targeted lesion in order to help the pathologist to make a correct diagnosis. 

The overall diagnostic accuracy of frozen section in our study was 90.9% supported by other previous studies which were 86.8%-95% (Mitra et al., 2010; Chand et al., 2016; Samal et al., 2017). There were 2 out of 22 cases over graded as high-grade glioma. This was attributed by the well-known freezing artefact in frozen section. The nuclear pleomorphism and nuclear membrane irregularities caused by freezing artefact were misinterpreted. Some of the other cases encountered difficulties during analysis due to similar reason. Mitosis were also difficult to identify as the cytomorphology of the tumour cells were hampered. Optimal temperature of frozen machine is required during the procedure to reduce the artefact. Features of necrosis and microvascular proliferation able to aid the pathologist in grading the glioma if other features were difficult to interpret. Study done by Obeidat et al., (2016) experienced similar problem and concluded that those features were reliable to overcome the problem. 

Moreover, the tissue thickness and staining quality play roles in making the diagnosis. These factors can affect the cellularity and cytoarchitectural assessment (Khoddami et al., 2015). Two of the cases were regard as high cellularity due to thickened section (>8 µm in thickness). The cytoarchitectural detail was difficult to interpret as the cells overlapped with each other and the tissue were folded. Nuclear hyperchromasia was not well visualized when the cells were not properly stained. This signified the important of competent lab personnel in handling the specimen to produce good quality slides. Sampling adequacy, quality slides preparation, heterogenicity of glioma, pathologist expertise, and communication between pathologists and surgeons are also the major contributing factors in determining the accuracy of frozen section in glioma. 

Based on our study, there was no significant difference in diagnostic accuracy of cytology smear and frozen section as both techniques revealed comparable and high diagnostic accuracy. Other studies also conclude similar findings (Ahmed et al., 2015; Nanarng et al., 2015; Louis and von Deimling, 2017; Agrawal et al., 2014). This provide an alternative solution to the centres that are lack frozen section facilities and skills. Furthermore, we found that cytology smear and frozen section were complimentary to each other. Few of our frozen sections failed to demonstrate the architectural detail and cytomorphology of glioma due to significant freezing artefact. Fortunately, this shortfall was overcome by the aid of cytology smear. We also experienced difficulties in diagnosing Pleomorphic Xanthroastrocytoma, WHO grade II on cytology smear. The hypercellular tumour cells exhibit nuclear atypia evidenced by nuclear pleomorphism, irregular nuclear membrane and some having multinucleation. Frozen section was able to help us to diagnose this entity. No vascular endothelial proliferation, necrosis or mitotic activity were visualized in our case. Clinical information and imaging assisted us during the pathological evaluation. It is recommended to use both techniques to improve the diagnostic accuracy if facilities and skills are available. 

In addition, complete clinical history, neuroimaging information, adequate sampling by the surgeon, good technical skills and trained neuropathology will help in achieving accurate and rapid diagnosis of glioma. Good communication between the surgeon and pathologist also plays a crucial in management of the patient. 

Our main limitation in this study was sample size. Total of 18 cases were not included due to unavailability of the cytology smears, frozen section and/or paraffin slides. Paraffin slide was unavailable in two of the cases due to tissue exhaustion. The surgeon cancelled some of the cases for intraoperative consultation as the surgeon were confident on the diagnosis and tumour grading based on clinical, imaging and intraoperative findings. Some of the cases that were clinically diagnosed as glioma turn out to be non-glial tumour during intraoperative consultation. We suggest expanding this study to other hospitals in Malaysia to obtain bigger sample size.
